# Pomegranate seed clustering by machine vision

**DOI:** 10.1002/fsn3.475

**Published:** 2017-11-12

**Authors:** Mohammad Reza Amiryousefi, Mohebbat Mohebbi, Ali Tehranifar

**Affiliations:** ^1^ Department of Food Science and Technology Neyshabur University of Medical Sciences Neyshabur Iran; ^2^ Department of Food Science and Technology Faculty of Agriculture Ferdowsi University of Mashhad Mashhad Iran; ^3^ Department of Horticultural Science Faculty of Agriculture Ferdowsi University of Mashhad Mashhad Iran

**Keywords:** Clustering, Image analysis, PCA, Pomegranate seed

## Abstract

Application of new procedures for reliable and fast recognition and classification of seeds in the agricultural industry is very important. Recent advances in computer image analysis made applicable the approach of automated quantitative analysis in order to group cultivars according to minor differences in seed traits that would be indiscernible in ocular inspection. In this work, in order to cluster 20 cultivars of pomegranate seed, nine image features and 21 physicochemical properties of them were extracted. The aim of this study was to evaluate if the information extracted from image of pomegranate seeds could be used instead of time‐consuming and partly expensive experiments of measuring their physicochemical properties. After data reduction with principal component analysis (PCA), different kinds of overlapping between these two types of data were controlled. The results showed that clustering base on all variables of image features contain more similar cultivars with clustering base on physicochemical properties (66.67% for cluster 1, 75% for cluster 2, and 50% for cluster 3). Therefore, by applying image analysis technique, the seeds almost were placed in different pomegranate clusters without spending time and additional costs.

## 1 INTRODUCTION

The pomegranate is native from Iran to the Himalayas in northern India, and has been cultivated and naturalized over the whole Mediterranean region since ancient times (Meerts et al., 2009). In Iran, pomegranate production and harvested area are over 700,000 tons per year and 56,000 ha, respectively (Eikani et al., 2012).

Pomegranate seed is a residue obtained from pomegranate juice and it contains vitamin E, sterols and 9c, 11t, 13c‐octadecatrienoic acid, called punicic acid, in good quantities. The seed content of the pomegranate yields an average amount of about 37–143 g/kg of fruit. It has been reported that pomegranate seed oil has a broad spectrum of biological activities, such as antioxidant and eicosanoid enzyme inhibition properties, suppressing chemically induced carcinogenesis, exerting antiangiogenic activity and immunomodulatory function. Pomegranate seed oil is considered as high‐quality oil recently touted for its health benefits (Eikani et al., 2011; Liu et al., 2009).

Since pomegranate consumption is driven by both fresh market and processing industry, it is crucial to acknowledge all fruit characteristics to not only classify varieties from a botanical point of view, but also to meet current market demand for quality fruits (Martínez, Melgarejo, Hernández, Salazar, & Martínez, 2006).

In order to recognize different kinds of pomegranate seeds, it is better to simulate the mechanism that occurs in ocular inspection. It means that grouping of the seeds should be based on knowledge of seed size, shape, and color.

Computer vision is the science that deals with object recognition and classification by extracting useful information about the object from its image or image set. The major tasks performed by a machine vision system can be grouped into three processes: image acquisition, processing or analysis, and recognition (Amiryousefi, Mohebbi, & Khodaiyan, 2014).

Currently, image analysis is a well‐established complement of morphology characterization. The image analysis technique allows the enhancement of images, as well as the identification and automatic isolation of particles for further study. In addition, it is a rapid and time‐saving technique that allows for the acquisition of quantitative data that could be very difficult or even impossible to obtain otherwise (Amaral, Rocha, Gonçalves, Ferreira, & Ferreira, 2009). Pixels are basic components of images. Two kinds of information are contained in each pixel, that is, brightness value and locations in the coordinates that are assigned to the images. The former is the color feature while features extracted from the latter are known as size or shape features (Zheng, Sun, & Zheng, 2006).

It is then of major technical and economical importance to implement computer‐based methods for reliable and fast identification and classification of seeds. Automatic systems can be based on seed images, from which classification features associated to seed morphological parameters and color are readily obtained. Thus, the field of machine vision, that is, image processing algorithms complemented with classification methods, seems a suitable framework for automatic seed identification (Granitto, Verdes, & Ceccatto, 2005). Besides, varietal identification of pomegranate is also of major interest in the horticultural industry.

Recent researches on the classification and identification of different grains by use of morphological or color features have been reported (Majumdar & Jayas, 2000; Nielsen, 2003; Paliwal, Borhan, & Jayas, 2004; Shouche, Rastogi, Bhagwat, & Sainis, 2001; Sokefeld, Gerhards, Kuhbauch, & Nabout, 1999; Tetsuka, Rotkiewicz, Kozirok, & Konopka, 2005; Utku, 2000).

During characterization processes, a large amount of data is usually obtained, therefore it becomes necessary for the use of statistical techniques to obtain accurate information about the seed characteristics. Multivariate analysis has traditionally been employed for food‐quality evaluation. PCA is a frequently employed statistical analysis and has been successfully applied for data reduction (Castell‐Palou, Rosselló, Femenia, & Simal, 2010; Kallithraka et al., 2001).

This study aimed to understand how much image features could be used in clustering of pomegranate seed. Therefore, clustering according to physicochemical features was first performed and then different types of image‐based clustering were matched.

## 2 MATERIALS AND METHODS

### 2.1 Sample preparation

Twenty fresh ripe pomegranate cultivars in commercial stage were harvested randomly in September 2009 from different mature trees (14 years old) to represent the population of the plantation from Agricultural Research Centre of Yazd province, Iran. The average temperature, the amount of rainfall, and relative humidity in growing season of 2009 were 28.65°C, 20 mm, and 26%, respectively. All cultivars were grown under the same geographical conditions and with the same applied agronomic practices.

The cultivars were: “Shirine Pust Sefeed” (SPS), “Malase Pust Nazok” (MPN), “Malase Save” (MS), “Vahshie Jangali Ghaemshahr” (VJG), “Shekarnare Pust Koloft” (SPK), “Mohalie Parand Gorgan” (MPG), “Malase Dane Siah Ramhormoz” (MDSiR), “Malase Dane Sefeed Ramhormoz” (MDSR), “Pust Sefeed Dezfol” (PSD), “Zaghe Yazdi” (ZY), “Garaje Shavar Yazdi” (GSY), “Pust Siah Abarndabad” (PSA), “Malase Mamoli Sarjo” (MMS), “Malase Porbar Sarvan” (MPS), “Khazare Bajestani” (KB), “Mazarie Bajestani” (MB), “Dom Ambaroti” (DA), “Shishe Kap” (SK), “Torshe Shahvar Ferdows” (TSF), and “Lilie Pust Koloft” (LPK).

Fruits were transported by a ventilated car to the laboratory as soon as harvested and defective pomegranates (sunburns, cracks, cuts, and bruises in peel) were discarded. The fruits were kept at 4°C until analysis.

### 2.2 Physicochemical properties

Physicochemical properties and antioxidant activity were determined on 20 fruits randomly selected from each cultivar. Fruit volume was measured by liquid displacement method. Fruit density was estimated by Westwood (1993).

Fruit length and diameter were measured by a digital vernier caliper with 0.01 mm sensitivity. The measurement of fruit length was made on the polar axis. The maximum width of the fruit, as measured in the direction perpendicular to the polar axis, is defined as the diameter. Arils were separated and total aril sand peel per fruit was measured as above. The peel thickness was measured by a digital vernier caliper. Fruit juice content was measured by extracting of total arils per fruit using an electric extractor (model 5020, Toshiba, Tokyo, Japan). The peel, aril, and juice percentage were calculated according to the method described by Zamani (1990).

After that, the major chemical compositions and antioxidant activity of pomegranate were analyzed.

The pH was determined with a digital pH meter (model 601, Metrohm, Herisau, Switzerland). Titratable acidity (TA) was characterized by titration to pH 8.1 with 0.1 N NaOH and presented as g of citric acid per 100 g of juice (AOAC, 1984).

Total soluble solid (TSS) was determined with a digital refractrometer (Erma, Tokyo, Japan). Total sugars were estimated according to the method described by Ranganna (2001), and ascorbic acid was determined by Ruck (1963).

Total anthocyanins were determined with the pH differential method (Giusti & Wrolstad, 2001) and the results were expressed as mg cyaniding‐3‐glucoside 100 g of juice. Total phenolics were measured colorimetrically at 760 nm using the Folin‐Ciocalteu reagent (Singleton & Rossi, 1965). The results were expressed as mg gallic acid equivalent in 100 g of juice.

Antioxidant activity was assessed according to the method of Brand‐Williams, Cuvelier, and Berset (1995).

Briefly, 100 μl of pomegranate juice diluted in the ratio of 1:100 with methanol: water (6:4, v/v) was mixed with 2 ml of 0.1 mmol/L 1,1‐diphenyl‐2‐pycrylhydrazyl (DPPH) in methanol. The mixtures were shaken vigorously and left to stand for 30 min. Absorbance of the resulting solution was measured at 517 nm by a UV‐visible spectrophotometer (model 2010, Cecil Instr. Ltd., Cambridge, UK). The reaction mixture without DPPH was used for the background correction. The antioxidant activity (AA) was determined by this relationship:

AA(%)=[1−(Abs Sample/Abs control)]×100

### 2.3 Image features

#### 2.3.1 Image acquisition

In the next stage, an image processing and analysis software was developed to determine the morphological parameters and color of pomegranate seeds. For this purpose, first the seeds were pretreated. Skin and other impurities were separated from pomegranate seeds, and the seeds were then washed with water and air‐dried.

The images were prepared using a flatbed scanner (HP ScanJet G4010, Hewlett Packard Co., CA, USA) with resolution of 600 dpi and the following settings: highlight 190, shadows 40, and midtones 1 (scanning software HP Precisionscan Pro, Hewlett Packard Co.). In each image acquisition, about 70 pomegranate seeds were placed on glass plate of the scanner avoiding seed to seed contact. The seeds were then covered by a nonreflecting black surface. All images were taken to approximately fill the scanner field of view and for further analysis, the images were stored in JPEG format.

#### 2.3.2 Image processing and feature extraction

**Figure 1:**
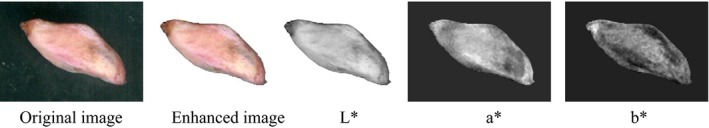
Schematic view of color measurement for a seed of MDSiR cultivar

For color determination, the contrast of images’ background were improved and manual segmentation were done (to extract the true images of pomegranate seeds from background) using Adobe Photoshop (Adobe, v.12.0). Since the L*a*b* color is device independent and providing consistent color regardless of the input or output (Yam & Papadakis, 2004), the preprocessed images were converted into L*a*b* units. Schematic view of color measurement for a seed of MDSiR cultivar is shown in Figure 1.

**Figure 2:**
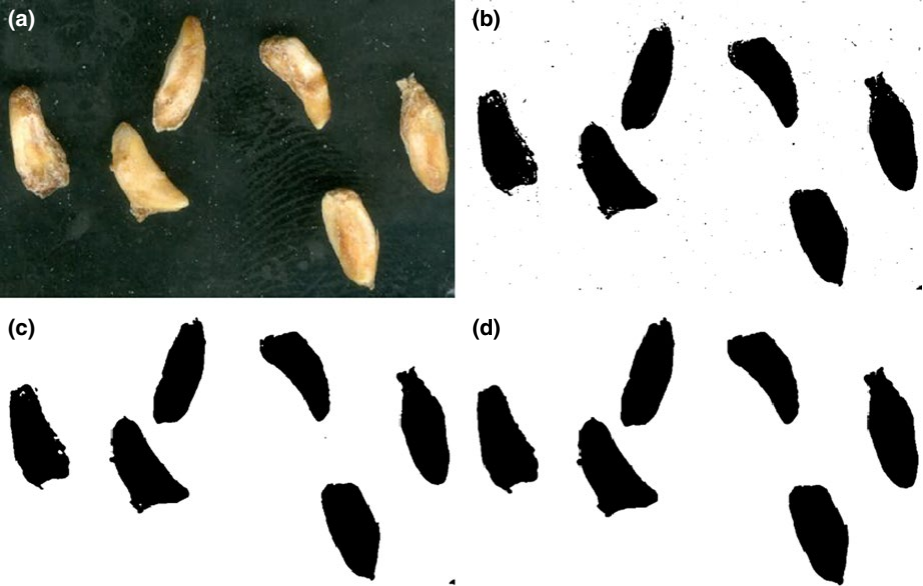
Schematic view of preparing images to determine the morphological parameters of VJG cultivar (a=original, b= make binary & threshold (Autso), c=median filter (r = 2 pixel), d=dilation)

The procedure of preparing images to determine the morphological parameters was different. Figure 2 depicts a schematic view of this procedure for six seeds of a typical variety (VJG).

As the binary images usually are used for detecting the particle information, after image acquisition using ImageJ software (National Institutes Health, Bethesda, Md, USA) version 1.45e, the images were converted to binary format.

The identification of each of the pomegranate seeds were performed by segmentation. Segmentation was accomplished using Otsu algorithm in Image J. The Otsu's threshold algorithm searches for the threshold that minimizes the intraclass variance, while the manual method assigns the threshold by finding each of the summits of the histogram of frequencies and then the bezels between them (Gonzales‐Barron and Butler, 2006; On‐line docs ImageJ software).

In Otsu's algorithm, the optimal threshold value (t*), expressed in terms of class probability (ω*i*) and class mean (μ*i*) can be obtained by a step sequence: (1) computing the probability of each intensity gray level (*pi*), (2) establishing the initial probabilities (ω*i*) and means (μ*i*), (3) stepping through all possible thresholds (*t *=* *1…maximum intensity) and (4) updating ω*i* and μ*i* to acquire the eligible threshold (t*) which corresponds to the maximum between‐class variance (Farrera‐Rebollo et al., 2011).

$$\left(\sigma_{B}^{2}\right)\left(t\right)=\frac{{[\mu_{T}\omega \left(t\right)-\mu \left(t)\right]}^{2}}{\omega (t)[1-\omega (t)]}$$

Where

$$\omega \left(t\right)=\sum_{i=0}^{t}p\left(i\right)$$

$$\mu_{\left(t\right)}=\sum_{i=0}^{t}ip\left(i\right)$$

$$\mu_{T}=\sum_{i=0}^{L-1}ip\left(i\right)\;\left(\text{Total mean of the whole image}\right)$$

The next step was reducing the effect of noise and outliers with median filter (*r* = 2 pixel). Afterward, dilation as one of the two basic operators in the area of mathematical morphology, applied to the filtered images. The basic effect of the operator on a binary image is to gradually magnify the boundaries of regions of foreground pixels. Thus, areas of foreground pixels enlarge in size while holes within those regions become smaller.

The enhanced images were acted to get a detailed explanation of the overall morphology. For each individual pomegranate seed, the acquired size parameters were *Area* (mean area of seeds in square pixels), *Perimeter* (the length of the outside boundary of the selection), *Minimum Feret Diameter* MFD (minimum distance between parallel tangents) and from the side view image, *Shape Descriptors* including *Circularity* (4π*area/perimeter^2), *Roundness* (4*area/(π*major axis^2)), and *Solidity* (area/convex area) (Rasband, 2006).

### 2.4 Principal component analysis

Principal component analysis (PCA), also known as Karhunen–Loeve transform, is extensively applied for dimensionality reduction, loss data compression, and feature extraction. This method projects the data orthogonally onto a lower dimensional linear space such that the variance of the projected data is maximized. Mathematically, PCs are linear transformations of the original measured set of variables. The calculation of PCs is actually a task of finding these indices of linear transformation. The principal difference between PCA and other types of linear transforms is that the transformation depends on the inherent structure of the data. The PCs are uncorrelated and ordered so that the first PC demonstrates the largest amount of variation and each successively defined PC expresses decreasing amount of variation. The first few PCs contain most of the variation in the original data set. The lack of correlation means that the PCs are measuring different dimensions in the data. The best results from PCA are obtained when the original variables are highly correlated (Chandraratne, Kulasiri, Frampton, Samarasinghe, & Bickerstaffe, 2006; Kokiopoulou and Saad, 2007).

In this study, PCA was used to reduce the dimensionality of the data. The reduced feature spaces were used for agglomerative hierarchical clustering. The analysis was performed with XLSTAT 2011 statistical package.

### 2.5 Clustering

Clustering methods can be divided into two basic types: hierarchical and partitional clustering. Within each of the types there exists a wealth of subtypes and various algorithms for finding the clusters. Hierarchical clustering proceeds successively by either merging smaller clusters into larger ones, or by splitting larger clusters. Partitional clustering, on the other hand, attempts to directly decompose the data set into a set of disjoint clusters (Rokach & Maimon, 2005).

Agglomerative hierarchical clustering (AHC) as one of the most popular clustering methods is defined by a stepwise algorithm which merges two objects at each step, the two which have the least dissimilarity. The algorithm first collects all the most similar observation pairs, then progressively stacks up the other observation groups until all the observations are in a single group. The AHC produces a binary clustering tree (dendrogram), whose root is the class that contains all the observations. This dendrogram represents a hierarchy of partitions, where a partition is obtained by truncating the dendrogram at a certain level. The partition contains fewer and fewer clusters as the truncation is made in the top of the dendrogram (i.e., toward the root). Clustering was performed using XLSTAT 2011 statistical package. XLSTAT proposes selected coefficients and criteria based on their mathematical properties and their practical or pedagogical interest. The dissimilarity between clusters of objects can be defined in several ways, called aggregation criteria, for example, the maximum dissimilarity (complete linkage), minimum dissimilarity (single linkage), average dissimilarity (average linkage) or Ward method (Addinsoft, 2007; Cotta & Moscato, 2003; Ghouila et al., 2009).

The method proposed by Ward (1963) aggregates two groups so that within‐group inertia increases as little as possible to keep the clusters homogeneous. In this study, based on the nature of data, this aggregation criterion having the least susceptibility to noise and outliers was applied. Ward's distance (*D*_*w*_) between clusters *C*_*i*_ and *C*_*j*_ is the difference between the total within‐cluster sum of squares for the two clusters separately, and the within‐cluster sum of squares resulting from merging the two clusters in cluster *C*_*ij*_:

$$D_{w}\left(C_{i},C_{j}\right)=\sum_{x\in C_{i}}{\left(x-r_{i}\right)}^{2}+\sum_{x\in C_{j}}{\left(x-r_{j}\right)}^{2}-\sum_{x\in C_{ij}}{\left(x-r_{ij}\right)}^{2}$$

where *r*_*i*_, *r*_*j*_, and *r*_*ij*_ are the centroids of *C*_*i*_, *C*_*j*_, and *C*_*ij*_, respectively.

## 3 RESULTS AND DISCUSSION

### 3.1 PCA outcomes

To achieve satisfactory results in a statistical multivariate analysis, the selection of variables should be carefully considered, so that only relevant variables must be included in the analysis. The results of the PCA for image features and physicochemical properties are presented in Table 1. The analysis demonstrates that 40.09% of the total variation in the image features is explained by the first PC, 64.56% by the first two PCs, 83.34% by the first three PCs, and the 94.96% by the first four PCs. That means 94.96% of the total variance in all the nine image features can be reduced into four PCs.

**Table 1:** Results of the PCA for image features and physicochemical properties

Principal components	Eigen value	% Variance	Cumulative variance %
Image features			
PC1	3.61	40.09	40.09
PC2	2.20	24.47	64.56
PC3	1.69	18.78	83.34
PC4	1.05	11.62	94.96
Physicochemical properties			
PC1	5.91	28.12	28.12
PC2	4.10	19.55	47.67
PC3	2.77	13.17	60.84
PC4	2.25	10.70	71.54
PC5	1.45	6.90	78.44
PC6	1.28	6.08	84.51

Also, the analysis of physicochemical properties shows that 28.12% of the total variation is explained by the first PC, 47.67% by the first two PCs, 60.84% by the first three PCs, 71.54% by the first four PCs, 78.44% by the first five PCs, and 84.51% by the first six PCs (Table 1). PCA allowed the reduction in the 21 variables into six PCs which explained 84.51% of the total variance.

### 3.2 Principal components loading

Principal components loading (eigenvectors) and correlations between variables and PCs of image features and physicochemical properties are shown in Tables 2 and 3, respectively.

**Table 2:** Eigenvectors (EV) and correlations (R) between variables and PCs of image features

Variable	PC1		PC2		PC3		PC4	
	EV	R	EV	R	EV	R	EV	R
1. Area	−0.28	−0.53	0.42	0.62	0.00	0.01	−0.53	−0.54
2. Perimeter	−0.48	−0.91	0.24	0.36	0.08	0.10	−0.12	−0.13
3. Circularity	0.50	0.95	−0.04	−0.07	−0.10	−0.13	−0.22	−0.23
4. Roundness	0.41	0.78	0.36	0.54	−0.09	−0.12	−0.04	−0.04
5. Solidity	0.45	0.86	−0.07	−0.10	−0.14	−0.18	−0.28	−0.29
6. MFD	0.12	0.23	0.63	0.93	−0.02	−0.02	−0.19	−0.20
7. L value	0.16	0.30	0.00	−0.01	0.72	0.94	−0.09	−0.09
8. a value	0.03	0.06	0.39	0.58	−0.37	−0.49	0.60	0.61
9. b value	−0.17	−0.31	−0.29	−0.43	−0.54	−0.71	−0.42	−0.43

**Table 3:** Eigenvectors and correlations between variables and PCs of physicochemical properties

Variable	PC1		PC2		PC3		PC4		PC5		PC6	
	EV	R	EV	R	EV	R	EV	R	EV	R	EV	R
1. Fruit length	0.12	0.28	0.40	0.82	−0.09	−0.14	−0.03	−0.05	0.08	0.10	0.14	0.16
2. Fruit diameter	0.05	0.13	0.46	0.94	0.03	0.05	−0.05	−0.08	−0.12	−0.15	0.09	0.11
3. Fruit volume	0.06	0.14	0.46	0.93	0.03	0.05	−0.13	−0.20	−0.14	−0.17	0.06	0.07
4. Fruit density	0.32	0.76	−0.19	−0.38	−0.13	−0.22	0.05	0.08	0.00	0.00	−0.13	−0.15
5. Calix length	−0.10	−0.25	0.13	0.27	0.16	0.26	0.12	0.18	−0.26	−0.31	0.20	0.23
6. Calix diameter	−0.21	−0.50	−0.01	−0.01	−0.16	−0.27	0.16	0.25	−0.45	−0.54	0.14	0.16
7. Thickness skin	−0.35	−0.84	0.14	0.28	−0.15	−0.25	−0.08	−0.13	−0.03	−0.03	0.13	0.15
8. Skin/fruit %	−0.39	−0.95	0.09	0.18	−0.04	−0.07	0.02	0.03	0.07	0.09	−0.05	−0.06
9. Aril/fruit %	0.40	0.96	−0.10	−0.20	0.03	0.06	−0.05	−0.07	−0.02	−0.03	0.03	0.03
10. Seed humidity weight	0.21	0.52	0.33	0.66	−0.12	−0.18	0.22	0.33	0.19	0.23	−0.14	−0.16
11. Seed/fruit %	0.23	0.55	0.15	0.30	−0.13	−0.22	0.33	0.50	0.32	0.39	−0.20	−0.23
12. Juice volume	0.33	0.81	0.12	0.24	0.07	0.12	−0.24	−0.37	−0.24	−0.29	0.12	0.13
13. Juice density	−0.05	−0.13	−0.16	−0.33	−0.22	−0.37	−0.20	−0.30	0.16	0.20	0.59	0.67
14. Juice fruit/fruit %	0.34	0.82	−0.19	−0.38	0.08	0.13	−0.17	−0.25	−0.18	−0.21	0.16	0.18
15. pH	0.08	0.19	0.07	0.14	0.51	0.85	0.20	0.30	0.00	−0.01	0.17	0.20
16. T.S.S	0.09	0.21	−0.01	−0.03	0.31	0.51	0.39	0.59	0.10	0.12	0.42	0.47
17. TA (mg.100 g)	0.11	0.26	−0.24	−0.49	−0.40	−0.66	0.21	0.32	−0.13	−0.15	0.08	0.09
18. Anthocyanin (mg.100 g)	0.19	0.46	0.12	0.24	−0.33	−0.55	−0.26	−0.39	0.14	0.17	0.30	0.34
19. Total phenolics (mg.100 g)	0.07	0.18	−0.05	−0.10	−0.13	−0.21	0.48	0.72	−0.36	−0.44	0.13	0.15
20. Total sugars (mg.100 g)	−0.10	−0.24	−0.06	−0.11	0.01	0.01	0.19	0.28	0.50	0.60	0.32	0.36
21. Antioxidant %	0.03	0.07	0.19	0.39	−0.42	−0.69	0.25	0.38	−0.07	−0.08	0.00	0.00

### 3.3 PC scores

Four new image features (PC scores) were measured as a linear combination of the features. For each sample, PC scores were calculated as the summation of the principal component loading multiplied by the respective measured variable. For example, PC scores for image features =∑ (−0.28 × Area −0.48 × Perimeter +0.50 × Circularity +0.41 × Roundness …).

Loading coefficients obtained from the application of PCA are useful for expressing the correlation between the original and the PCA‐transformed variables. The higher the weighting, the more the variables have in common with the PC and the more it contributes to what the PC explains of the data structure. For example, in the case of image features, PC1 was high in circularity (0.95), roundness (0.78), and solidity (0.86) with positives values; and also high in perimeter (0.91), but with negative value. PC2 was high in minimum Feret diameter (0.93), and PC3 was high in L value (0.94), with positive values. Also, six PC scores were calculated as linear combinations of measured physicochemical properties.

### 3.4 PC indicators

The other alternative to PC scores is that the most correlated measured variable be selected to represent PCs (PC indicator). This is computationally attractive, as there is no need to extract all the variables. Only the selected variables can be extracted (Chandraratne et al., 2006). The four image features selected for PC indicator are: Circularity, Minimum Feret Diameter, L*, and a* parameters.

Meanwhile, the six physicochemical properties selected for PC indicator are: fruit diameter, % aril/fruit, juice density, total sugars, total phenolics, and pH.

### 3.5 Clustering results and overlapping of them

All variables, PC scores, and PC indicators were used for clustering. Results of clustering based on different variables and the cultivars exposure in each cluster are shown in Table 4.

**Table 4:** Results of agglomerative hierarchical clustering (AHC) based on different variables

Clustering base	Variable	Cluster no.	Cultivars	Objects	Within‐class variance	Average distance to centroid
Image features	All variables	1	SPS, MPN, SPK, PSD, PSA, DA	6	9.69E + 04	2.51E + 02
		2	MS, VJG, ZY, GSY, MMS, KB, MB, SK, TSF, LPK	10	2.64E + 04	1.20E + 02
		3	MPG, MDSiR, MDSR, MPS	4	1.08E + 05	2.42E + 02
	PC scores	1	SPS, VJG, MPG, MDSR,PSD, MPS, DA	7	3.02E + 04	1.31E + 02
		2	MPN, SPK, MDSiR, PSA	4	1.50E + 04	9.77E + 01
		3	MS, ZY, GSY, MMS, KB, MB, SK, TSF, LPK	9	2.10E + 04	1.01E + 02
	PC indicators	1	SPS, MPG, ZY, SK, TSF	5	6.73E + 00	2.20E + 00
		2	MPN, VJG, SPK, PSD, GSY, PSA, DA, LPK	8	4.85E + 00	1.86E + 00
		3	MS, MDSiR, MDSR, MMS, MPS, KB, MB	7	1.50E + 01	3.46E + 00
Physicochemical traits	All variables	1	SPS, MS, MDSR, PSA, MMS	5	1.20E+07	3.02E + 03
		2	MPN, VJG, SPK, MPG, PSD, ZY, GSY, DA,SK, TSF, LPK	11	5.04E + 06	1.88E + 03
		3	MDSiR, MPS, KB, MB	4	6.09E + 06	1.85E + 03
	PC scores	1	SPS, MS, KB, TSF, LPK	5	2.42E + 06	1.31E + 03
		2	MPN, SPK, MPG, MDSiR, MDSR, GSY, PSA, MMS, MPS, MB	10	1.52E + 06	1.14E + 03
		3	VJG, PSD, ZY, DA, SK	5	1.82E + 06	1.16E + 03
	PC indicators	1	SPS, MPN, SPK, MDSR,GSY, PSA	6	3.06E + 06	1.19E + 03
		2	MS, VJG, PSD, ZY, DA, SK, TSF, LPK	8	1.11E + 06	8.18E + 02
		3	MPG, MDSiR, MMS, MPS, KB, MB	6	2.09E + 06	9.84E + 02

The maximum cultivars in one cluster are 11, and each cluster at least contains four cultivars. In order to evaluate how much image‐based clustering could be used for clustering of different cultivars of pomegranate seed, overlapping of the image‐based clusters with the results of clustering based on physicochemical properties were analyzed. The results are reported in Table 5.

**Table 5:** The percentage of overlapping for image‐based clustering and physicochemical‐based clustering

Image‐based clustering	Physicochemical‐based clustering	Cluster 1	Cluster 2	Cluster 3
PC indicators	All variables	20%	63.64%	100%
PC indicators	PC scores	40%	40%	0%
PC indicators	PC indicators	16.67%	50%	83.33%
PC scores	All variables	40%	18.18%	50%
PC scores	PC scores	20%	40%	40%
PC scores	PC indicators	33.33%	0%	50%
All variables	All variables	40%	54.55%	50%
All variables	PC scores	20%	30%	0%
All variables	PC indicators	**66.67%**	**75%**	**50%**

Clusters based on all variables of image features were composed of 6 (SPS, MPN, SPK, PSD, PSA, and DA), 10 (MS, VJG, ZY, GSY, MMS, KB, MB, SK, TSF, and LPK), and 4 cultivars (MPG, MDSiR, MDSR, and MPS), while, clustering of the PC indicators of physicochemical properties resulted six (SPS, MPN, SPK, MDSR, GSY, PSA), eight (MS, VJG, PSD, ZY, DA, SK, TSF, LPK), and six cultivars (MPG, MDSiR, MMS, MPS, KB, MB). As we see in Table 5, when overlapping of all variables of image‐based clustering with PC indicators of physicochemical‐based clustering were evaluated, the best result has been obtained (66.67% for cluster 1, including SPS, MPN, SPK, and PSA cultivars; 75% for cluster 2, including MS, VJG, ZY, SK, TSF, and LPK cultivars; and 50% for cluster 3, including MPG, MDSiR, and MPS cultivars). Although, the result of overlapping between PC indicators of image based clustering with all variables of physicochemical based clustering to some extent is acceptable. It means that based on the features extracted from pomegranate seed images and considering the physicochemical properties of them, the seeds successfully were placed in different pomegranate clusters with an acceptable degree of error. In addition, by this method time and cost could be saved.

**Figure 3:**
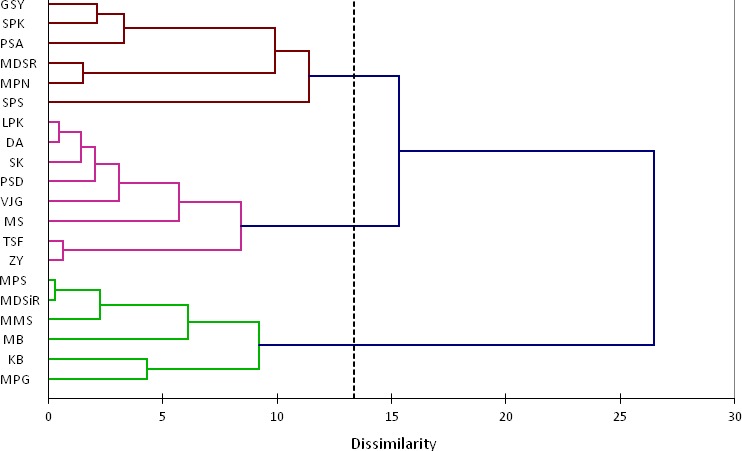
Dendrogram from hierarchical clustering of the PC indicators of physicochemical properties which groups 20 pomegranate seed cultivars

**Figure 4:**
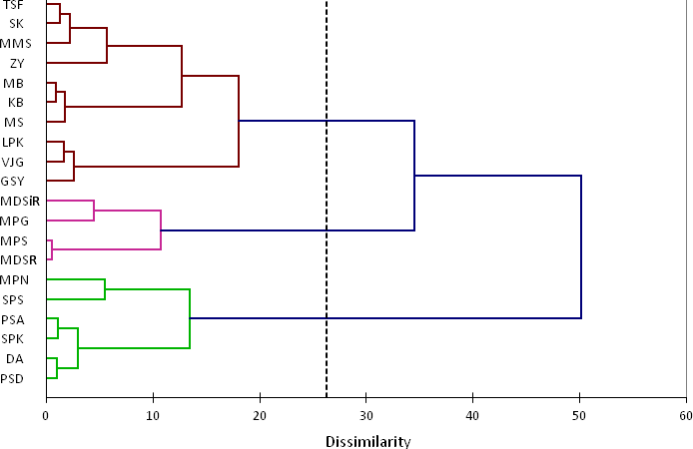
Dendrogram from hierarchical clustering of all variables of image features which groups 20 pomegranate seed cultivars

Clustering dendrogram from hierarchical clustering of the PC indicators of physicochemical properties and all variables of image features are given in Figures 3 and 4.

In these two dendrograms (Figures 3 and 4) it could be seen that how the algorithm of AHC progressively grouped the different pomegranate seed cultivars based on PC indicators of their physicochemical properties (Figure 3) and also all variables of the image features (Figure 4).

## 4 CONCLUSIONS

In this work, in order to cluster 20 cultivars of pomegranate seed, 9 image features and 21 physicochemical properties of them were extracted.

Application of PCA allowed the detection of the most important factors of variability according to the image features and physicochemical properties of the different pomegranate seeds. The results showed that clustering base on all variables of image features contain more similar cultivars with clustering base on physicochemical properties (66.67% for cluster 1, 75% for cluster 2, and 50% for cluster 3). Therefore, it could be concluding that it is possible to apply the information extracted from image of pomegranate seeds instead of time‐consuming and partly expensive experiments of measuring physicochemical properties of them.

## CONFLICT OF INTEREST

None declared.
